# Hysteria in Children

**Published:** 1905-07-22

**Authors:** 


### HYSTERIA IN CHILDREN.

Hysteria, although not common in childhood, would be more commonly found if it were more frequently looked for. It is really rare in children below the age of ten or eleven, and in early life does not often assume the gross forms associated with it in the years of later childhood and of early adolescence. But the series of cases collected by Dr. W. H. Summons[1] shows that even in children the disease may be almost as protean as it is in older subjects, although such frank evidences as hystero-epilepsy are seldom met with. The bulk of cases at this early period of life are either paralytic, or simulate the joint and bone lesions so commonly contemporaneous. These functional disorders of joints occasionally lead to grave contractures, and thus the early recognition of them becomes a point of high importance. The motor and convulsive symptoms include various forms of *tic*, resulting either in facial spasm or in torticollis. A common form assumed by the condition is that of chronic cough, which may be severe enough to suggest a serious lesion. This cough is often perpetuated by catarrhal conditions of the nasopharynx, especially those associated with adenoid vegetations.

Although the existence of hysteria in early life requires full recognition, it is important to remember that certain intracranial lesions particularly connected with this epoch may give rise to symptoms very highly suggestive of hysteria. Of these the most important is cerebral tumour. Cerebral tumours composed of tuberculous deposits are quite frequent in children, and not uncommonly give rise to passing attacks of tremor apart from any other symptom of disease. We may record in this connection the case of a boy of five, the only child of neurotic parents, who was subject to such occasional attacks of general tremor. He was admitted to a hospital, and a careful examination, which included investigation of the ocular fundus, revealed no sign of disease. During his residence in the hospital he was practically free from the attacks of trembling, whereas his parents reported them as frequent. Ultimately, in view of all the circumstances, a diagnosis of hysteria was reached by a process of exclusion, only to be reversed by the death of the patient not long after. Post-mortem examination revealed a tumour of the brain, whose only expression during life had been the temporary attacks of tremor above described.

[1] Intercolonial Jour. of Australasia, May 1905.

